# Symptoms and causes of obstacles in AI-based telemonitoring: strategies for system development through a CRISP-DM lens

**DOI:** 10.3389/frai.2026.1850272

**Published:** 2026-06-26

**Authors:** Sophie Haas, Jonas Hammer, Fabia Marie Hettler, Patricia Kajüter Rodrigues, Frank Teuteberg, Oliver Thomas

**Affiliations:** 1 Information Management and Information Systems, Osnabrueck University, Osnabrueck, Germany; 2 Smart Enterprise Engineering, German Research Center for Artificial Intelligence, Osnabrueck, Germany; 3 Accounting and Information Systems, Osnabrueck University, Osnabrueck, Germany

**Keywords:** cardiovascular disease, clinical decision support system, data governance, healthcare integration, regulatory challenges, remote monitoring, risk assessment, trustworthy AI

## Abstract

**Introduction:**

Chronic diseases, particularly heart failure (HF), impose a substantial clinical and socioeconomic burden worldwide. AI-based telemonitoring holds significant promise for improving chronic disease management through continuous monitoring and early detection of deterioration. However, translation into routine clinical practice remains limited due to challenges regarding data quality, transparency, regulation, and integration into established clinical workflows. Furthermore, the underlying end-to-end development processes are often inadequately documented and exhibit a positive publication bias, thereby limiting the opportunity to learn from prior efforts.

**Methods:**

This study employs a holistic, process-oriented approach to examining obstacles across the full development lifecycle of AI-based telemonitoring systems. Existing Cross Industry Standard Process for Data Mining (CRISP-DM) variants are synthesized into a structuring framework tailored to telemonitoring contexts and used to guide a qualitative case study of the KardioInterakt project, a German research initiative developing an AI-based telemonitoring system for HF management.

**Results:**

By systematically reconstructing decisions, constraints, and mitigation strategies and synthesizing them with prior work, we offer practice-oriented recommendations with potential transferability for the development of AI-based telemonitoring systems. These pertain to scoping and feasibility assessment, stakeholder involvement, data strategy, regulatory compliance, user-centered design, and transparent publication.

**Discussion:**

Our findings offer actionable guidance for the development of viable, trustworthy, robust, and regulatory-compliant AI-based telemonitoring solutions. The process-oriented recommendations address prevailing challenges and demonstrate how these can be managed across the development lifecycle, thereby promoting transparency and supporting successful integration into routine clinical practice.

## 1 Introduction

Cardiovascular diseases like chronic heart failure (HF) are the leading cause of morbidity and mortality worldwide, accounting for nearly 18 million deaths annually (World Health Organization, 2023). HF represents a major clinical and socioeconomic challenge due to its progressive nature, frequent (re-)hospitalizations, and high mortality rates. In recent years, digital health technologies like telemonitoring systems have emerged as promising tools to improve disease management, enable early detection of deterioration, and enhance patient outcomes (Koehler et al., 2018; Bachtiger et al., 2020). The increasing availability of wearable sensors like smartwatches or biosensor patches as well as continuous patient data streams provides an unprecedented opportunity for artificial intelligence (AI)-driven models to support clinical decision-making and personalized interventions (Johnson et al., 2018; Topol, 2019).

Despite a growing body of research on AI-based disease classification and risk prediction, many models fail to translate into real-world clinical settings due to challenges related to data quality, explainability, regulatory compliance, and clinical integration (Kelly et al., 2019; Reddy and Shaikh, 2025). Particularly, the systematic development process of such AI models, ranging from data acquisition to preprocessing, model selection, evaluation, and deployment, is often insufficiently documented or follows *ad hoc* procedures that impede reproducibility and regulatory validation (Studer et al., 2021; Pinsky et al., 2024). This shows that while the technical performance of AI models in cardiology and in HF in particular is well-studied, methodological frameworks for their development, evaluation, and operationalization in telemonitoring contexts remain underexplored (Bertl et al., 2023).

To address this research gap, the present study adopts a process-oriented perspective by synthesizing multiple variants of the Cross Industry Standard Process for Data Mining (CRISP-DM; Wirth and Hipp, 2000) into a unified framework tailored to the specific requirements of AI-based telemonitoring. By integrating complementary extensions highlighting medical, infrastructural, machine learning, and trust-related aspects into the baseline variant, the process model is adapted to the clinical, regulatory, and operational constraints of telemonitoring contexts. Subsequently, this synthesized framework is employed to systematically analyze development challenges and derive practical mitigation strategies in the case study of the KardioInterakt project. KardioInterakt is a German research initiative to develop a telemedical early-warning system designed for the continuous monitoring of patients suffering from chronic HF and the detection of clinical deterioration. This case-based application complements prior efforts that have primarily identified barriers and recommendations through literature reviews (e.g., Reddy and Shaikh, 2025), surveys (e.g., Zemplényi et al., 2023), and expert interviews (e.g., Nair et al., 2024). It should be noted that the identified challenges are not entirely novel or exclusive to AI-based systems, particularly regarding organizational coordination, clinical workflows, infrastructure, regulation, and stakeholder alignment. However, the integration of AI may amplify these challenges by introducing additional dependencies, for instance related to data availability and quality, model validation, trust, and monitoring. The contribution therefore lies in a domain-specific and process-oriented perspective on AI-based telemonitoring by linking recurring barriers to specific development phases and illustrating them with practical examples from a real-world use case, which may particularly aid less experienced researchers and developers in anticipating recurring pitfalls and avoiding preventable mistakes. Based on this, the following three research questions (RQs) can be derived:

*RQ1*: Which CRISP-DM phases and tasks are applicable to, and need to be adapted for the development of AI-based telemonitoring systems?

*RQ2*: Which methodological, regulatory, and practical obstacles and causes arise in CRISP-DM guided end-to-end development of AI-based telemonitoring solutions?

*RQ3*: Which mitigation strategies can be derived to address these obstacles, and how may they be operationalized as actionable development guidance?

This paper is organized as follows: We provide a comprehensive overview of related work on AI-based telemonitoring for HF, encompassing data modalities, system architectures, and regulatory constraints. In addition, we consider established variants of the CRISP-DM framework as conceptual basis for the development of AI systems in regulated telemonitoring contexts. These perspectives are integrated with empirical insights from KardioInterakt using a qualitative, process-oriented case study design that reconstructs key decisions, assumptions, and constraints across all CRISP-DM phases. Drawing from these empirical insights and synthesizing them with prior work, we derive case-based, potentially transferable lessons and actionable recommendations for the development of AI-supported health applications in real-world telemonitoring contexts.

## 2 Related work

### 2.1 State of the art in AI-based telemonitoring

Across medical fields, the use of AI for live monitoring of patient data has expanded considerably. In cardiovascular care, AI-enhanced telemonitoring supports early detection of arrhythmias and the management of chronic conditions such as HF (Gaoudam et al., 2025). Wearable devices with FDA-cleared algorithms (e.g., Apple Watch) continuously analyze photoplethysmography or electrocardiogram (ECG) signals and can flag atrial fibrillation with high sensitivity (Gaoudam et al., 2025). In metabolic diseases, continuous glucose monitors combined with AI models enable the prediction of glucose excursions and anticipatory insulin dosing in diabetes and prediabetes (Fraser et al., 2025).

Typical data sources and monitoring processes in AI-based telemonitoring include wearable ECG patches, wristbands or smartwatches for heart rate and rhythm, blood pressure cuffs, pulse oximeters, weight scales, and accelerometers for activity (Hinrichs et al., 2024). In HF telemonitoring, monomodal use cases often rely on vital-sign time series, whereas multimodal systems additionally integrate laboratory markers and clinical metadata to improve decompensation prediction and risk stratification (Shumba et al., 2023; Victor et al., 2024). For instance, in the TIM-HF2 setting, HF patients record daily weight, blood pressure, heart rate, single-lead ECG, and peripheral oxygen saturation, as well as scale-based wellbeing, which are transmitted to a telemedical center and used to trigger clinical actions (Hinrichs et al., 2024). Many recent systems enrich this pipeline with patient-reported information (e.g., symptom diaries, medication adherence) and selected electronic health record (EHR) data (Saha et al., 2025). This results in multimodal data streams that span structured EHR variables (diagnoses, medications, laboratory values), unstructured clinical text, and high-frequency sensor signals from wearables and stationary monitors. More specifically, unstructured information is estimated to represent about 80% of medical data and thus constitutes a large but technically demanding resource for AI pipelines (Kong, 2019; Sedlakova et al., 2023). Consequently, structured steps are required for data ingestion, cleaning (e.g., motion artefact removal), feature extraction, and temporal aggregation before model training and inference (Gaoudam et al., 2025).

For supervised learning, both data volume and temporal granularity are critical. Recent HF readmission models are therefore typically trained on large longitudinal cohorts; for example, Zhang et al. (2023) used data from 14,843 HF patients, collected over 10 years to develop a 30-day readmission model, illustrating the scale often required, whereas small prospective datasets rarely support complex models. In this context, data provenance is tightly linked to ethical and regulatory constraints. Regardless of whether data come from routine care, telemonitoring programs, or clinical trials, AI development usually requires ethics approval, informed consent, and compliance with the GDPR. If the system qualifies as a medical device, additional requirements from the MDR and the forthcoming EU AI Act apply (European Union, 2024; Vardas et al., 2025).

From a systems and tooling perspective, several works describe similar multi-tier architectures. At the edge, smartphone apps or home hubs aggregate sensor data in real time, while backend services handle storage, preprocessing, and analytics (Shumba et al., 2023; Manta et al., 2024). IoT platforms, cloud infrastructures, distributed databases, and RESTful APIs are commonly used to support continuous data streams from large patient cohorts (Manta et al., 2024). Interoperability with clinical systems is typically realized via HL7 FHIR-based data stores and APIs, as illustrated by the RETENTION architecture for HF, which defines separate layers for patient-side collection, site-level processing and a global analytics tier (Manta et al., 2024). Security and privacy are addressed through encryption, role-based access control, and pseudonymization (Manta et al., 2024).

For model development and deployment, related work mostly relies on standard machine-learning and deep-learning frameworks such as TensorFlow and PyTorch, together with libraries for time-series analysis (Shumba et al., 2023). In HF telemonitoring, expert systems are increasingly combined with models that exploit longitudinal vital signs and patient-reported data, as in the machine-learning-enhanced expert system for HF decompensation proposed by Saha et al. (2025). When deploying on resource-constrained devices, tinyML toolchains such as TensorFlow Lite or ARM’s CMSIS-NN are used to compress and optimize models for wearables and edge hardware (Shumba et al., 2023). Scoping reviews emphasize that streaming analytics, real-time inference, and, prospectively, online learning and periodic model retraining will be important to maintain calibration in changing populations (Abedi et al., 2025).

A further strand of work addresses clinical portals and decision support. Many telemonitoring platforms provide dashboards where clinicians review incoming data and AI-generated alerts, often integrated into hospital information systems via HL7/FHIR or SMART-on-FHIR interfaces (Manta et al., 2024). These systems implement rule-based triage logic or use model predictions to suggest follow-up actions. However, scoping reviews on AI-driven cardiovascular monitoring indicate that many studies do not fully report operational deployment aspects, such as latency, scalability, and mechanisms for ongoing model monitoring, highlighting a gap in best-practice frameworks for the deployment of real-time AI systems (Shumba et al., 2023; Abedi et al., 2025). This paper addresses this gap by deriving potentially transferable and actionable recommendations for the end-to-end development of AI-based telemonitoring systems, specifically for HF management.

### 2.2 CRISP-DM variants for AI-based telemonitoring

Methodological descriptions for AI projects typically address feature engineering, cross-validation, and internal or external validation (Hinrichs et al., 2024; Gaoudam et al., 2025; Saha et al., 2025), but rarely adopt process models to systematically reflect on challenges across all phases, from *Business Understanding* to *Monitoring and Maintenance* (e.g., Zemplényi et al., 2023; Nair et al., 2024). CRISP-DM is one of the most established process models for data mining projects and provides a clear phase-based structure for iterative development (Wirth and Hipp, 2000). However, AI-based telemonitoring in healthcare additionally requires explicit consideration of clinical and real-world implementation constraints (Lekadir et al., 2025), ML-specific validation, risk assessment, and lifecycle governance (Arnaout et al., 2024; Lekadir et al., 2025), as well as ongoing post-deployment monitoring and evaluation during operation (Arnaout et al., 2024). Therefore, we used CRISP-DM as the reference structure and complemented it with established extensions that address these additional requirements. Suitable CRISP-DM variants were identified through a targeted literature review, including relevant review articles and forward and backward searches (e.g., Shimaoka et al., 2024). The selection was based on the alignment with the objective of this study: analyzing obstacles in an AI-based telemonitoring setting across development and operational phases. Selection criteria were (1) clear proximity to CRISP-DM, (2) explicit additions relevant to AI-based telemonitoring (e.g., operational monitoring, governance, domain constraints), and (3) sufficient process detail to allow phase mapping. Table 1 summarizes the included CRISP-DM variants and their respective contributions to the synthesized framework.

**Table 1:** CRISP-DM variants, authors, and rationale for inclusion.

CRISP-DM variant	Author(s)	Rationale for inclusion
CRISP-DM 1.0	Wirth and Hipp (2000)	Provides the baseline phases for data-driven projects (reference model).
CRISP-MED-DM	Niakšu (2015)	A medical-domain extension that explicitly maps typical medical analytics challenges (e.g., data heterogeneity, clinical validity) onto a CRISP-DM-like structure.
DMME-CRISP-DM	Wiemer et al. (2019)	A holistic extension originating from engineering contexts (Data Mining Methodology for Engineering), highlighting data acquisition/infrastructure and system integration aspects relevant for sensor- and pipeline-driven telemonitoring.
CRISP-ML(Q)	Studer et al. (2021)	An ML-specific extension emphasizing quality assurance, risk awareness, and explicit lifecycle considerations such as monitoring and maintenance.
Trustworthy-AI procedure model aligned with CRISP-DM	Kortum et al. (2022)	Integrates Trustworthy-AI requirements and extends the process perspective toward development and operation of AI systems, which is particularly relevant for safety- and compliance-critical healthcare AI.

## 3 Methodology

This study examines recurring challenges, and underlying causes in the development of AI-based telemonitoring systems and derives mitigation strategies to support future system development. Telemonitoring should be conceptualized as a multi-stage care process, since its implementation relies on the interaction of data collection, transmission, analysis, and clinical use; consequently, barriers tend to emerge across these interconnected stages rather than within a single isolated technical step (Claggett et al., 2024; Liu et al., 2024). To capture these dynamics, we employed a process-oriented lifecycle lens and applied it to a retrospective case study.

Methodologically, the paper follows a two-step approach. First, we developed a conceptual synthesis of CRISP-DM and selected variants to derive a process model suitable for AI-based telemonitoring. Second, we conducted a retrospective single-case study of the KardioInterakt project: we mapped empirical observations (i.e., symptoms) to the synthesized phases and, for each phase, derived higher-level obstacles and associated causes. Finally, we consolidated recurring patterns across phases into a cross-phase synthesis, linking symptoms, causes, and obstacles, and developed mitigation strategies to highlight interdependencies and recurring lifecycle-spanning challenges. The empirical part is designed as a single-case study because this research strategy allows an in-depth examination of complex, context-dependent phenomena in real-life settings and is particularly suited to addressing “how” and “why” questions (Yin, 2018).

**Figure 1:**
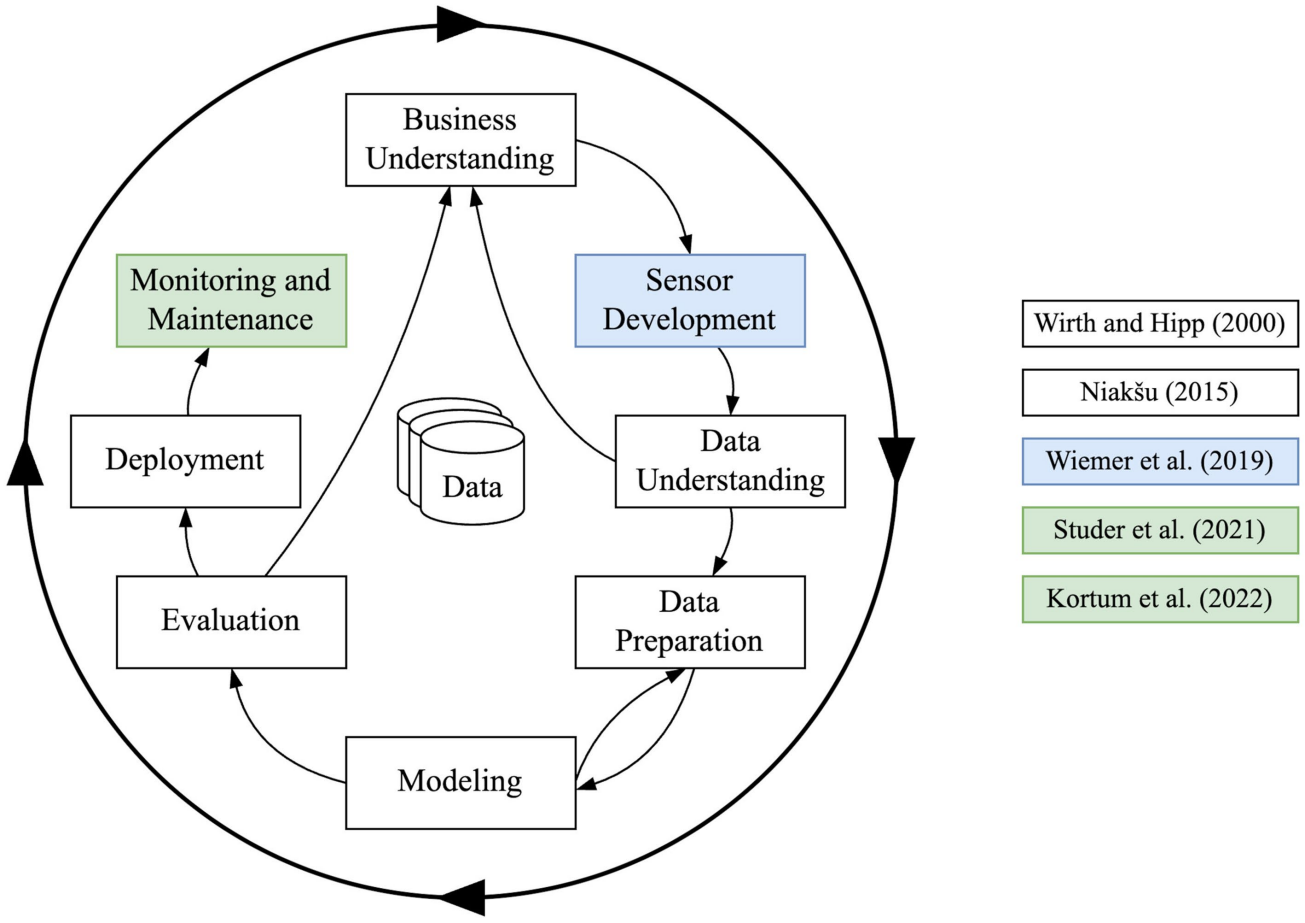
Combined CRISP-DM phases from different variants.

First, we conducted a comparative phase mapping across the five selected CRISP-DM variants. Starting from CRISP-DM 1.0 (Wirth and Hipp, 2000) as the baseline, we aligned semantically equivalent phases and retained additional phases if they added distinct lifecycle coverage relevant to AI-based telemonitoring (e.g., implementation engineering tasks and continuous operational monitoring). The synthesis resulted in a combined, iterative phase model comprising eight phases: *Business Understanding*, *Sensor Development*, *Data Understanding*, *Data Preparation*, *Modeling*, *Evaluation*, *Deployment*, and *Monitoring and Maintenance*. Depending on the specific telemonitoring use case, the *Sensor Development* phase may be considered optional in parts or in its entirety. Figure 1 visualizes the integrated phase model and indicates how phases are informed by the selected variants.

The empirical case is the KardioInterakt project (08/2022 to 01/2026), a multidisciplinary research project, funded by the German Federal Ministry of Research, Technology and Space. Its goal was to improve follow-up care for patients with chronic HF through digitally supported, contact-reduced aftercare. To this end, the project developed a telemonitoring system that continuously records patients’ vital parameters and relevant biomarkers via a novel wearable and transmits them to a shared web portal. Patients are monitored at home, while clinicians can remotely access their health data and intervene if signs of clinical deterioration emerge. By combining sensor technology, telemetric communication, and AI-based predictive analytics, KardioInterakt aims to detect early warning signs, reduce avoidable hospital readmissions, and support timely, personalized treatment decisions. In line with this objective, the project seeks to enhance patients’ quality of life, decrease hospitalization rates, and enable more efficient, digitally supported aftercare. To achieve this, the consortium comprised six partners contributing complementary institutional perspectives from university-based eHealth and socio-technical research, applied AI and information systems research, IT and strategy consulting, clinical heart failure care, industrial sensor development, and medical association expertise in cross-sectoral care communication. The authors were primarily involved in requirements analysis, process analysis, socio-technical evaluation, and AI-based platform development. T the project roadmap was initially planned for 36 months and centered around three partly overlapping phases: functional specification of the system with a planned completion time of 12 months; implementation of the AI-based interaction platform and smart patches, starting before completion of the first phase and planned for 18 months; and evaluation and transfer of results, starting before completion of implementation and planned for 12 months. The project was subsequently extended to a total duration of 42 months to compensate for delays in implementation and evaluation. In the present study, KardioInterakt serves as the empirical case for applying the synthesized CRISP-DM framework to the development and evaluation of an AI-based telemonitoring pipeline and reflecting on the encountered challenges.

The case analysis draws on project-internal development evidence generated during the KardioInterakt lifecycle (e.g., project documentation and iterative technical and clinical alignment), using the eight phases of the synthesized CRISP-DM model as a structuring framework. Following a case-study logic (Yin, 2018), we reconstructed phase-specific activities and consolidated evidence into an explanatory account of recurring development obstacles. For each phase, we identified symptoms (i.e., observable indications of friction, misfit, delays, rework, or failure modes) and consolidated them into overarching obstacles across phases (i.e., development barriers reflected by symptoms) and associated causes (e.g., resource constraints, unclear objectives, data limitations, governance gaps). The identification and consolidation of symptoms, obstacles, and causes was conducted in a series of author workshops. To enhance methodological rigor, the reconstruction combined project documentation, phase-specific reflections, interdisciplinary discussion, and comparison with prior research. As external validation was beyond the scope of this single-case study, preliminary mappings were reviewed and refined by project members with clinical, technical, and methodological expertise to reduce individual interpretive bias and ensure the synthesis remained grounded in the empirical case. Observations were iteratively assigned to CRISP-DM phases, discussed until consensus was reached, and consolidated into a cross-phase synthesis table. Mitigation strategies were derived as an interpretive synthesis grounded in project experience and complemented with supporting literature where applicable.

## 4 Results

**Figure 2:**
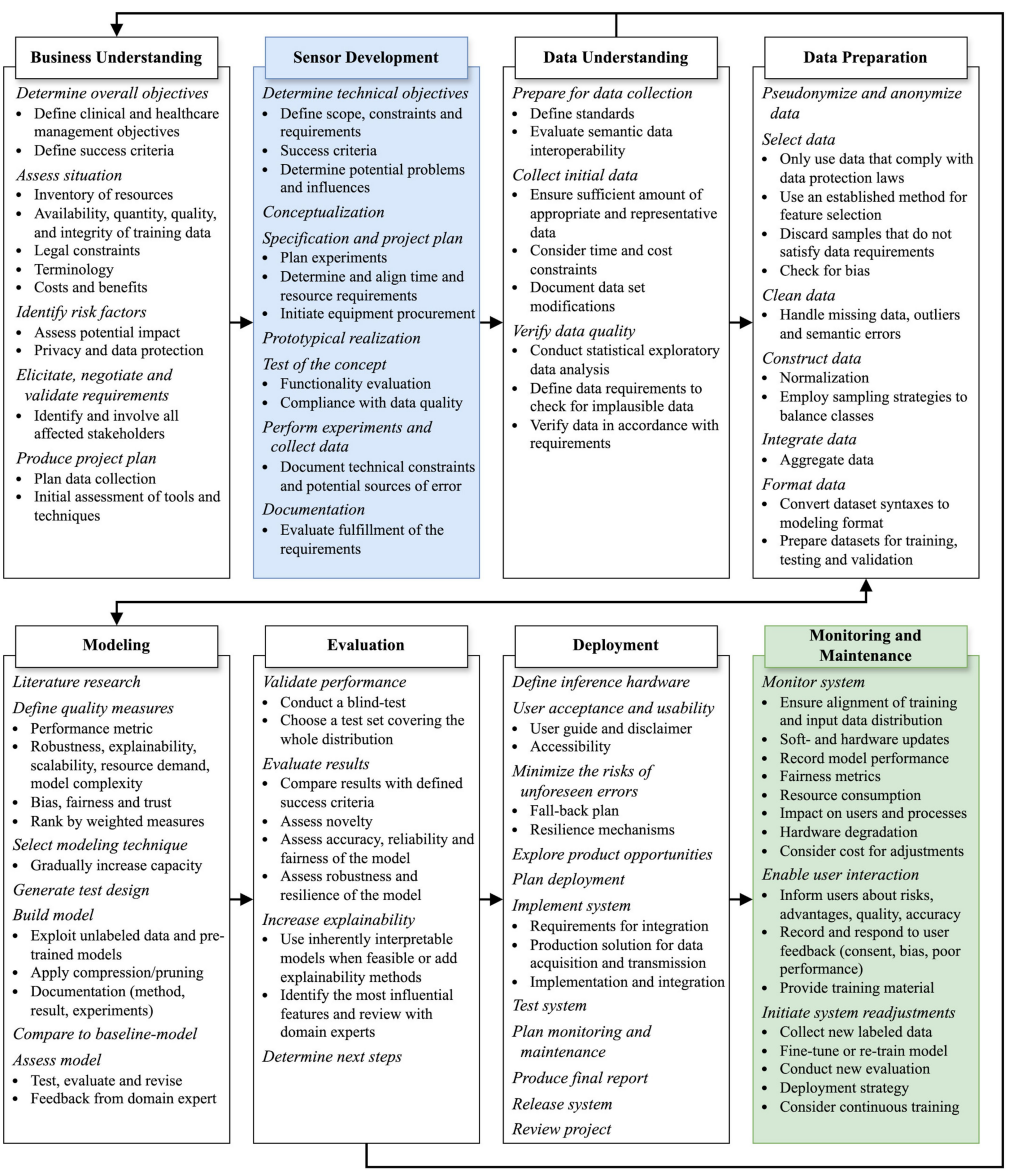
Synthesized CRISP-DM framework for AI-based telemonitoring contexts and associated tasks (with colors corresponding to Figure 1).

An overview of the synthesized process model, including the consolidated tasks derived from the different CRISP-DM variants, is provided in Figure 2. The subsequent sections provide a detailed composition of the associated tasks for each phase. In accordance with this task structure, the courses of action and the challenges, i.e., symptoms (S) encountered in KardioInterakt are described. Finally, the challenges are conceptualized as overarching obstacles, with associated causes and mitigation strategies.

### 4.1 Business understanding

**Figure 3:**
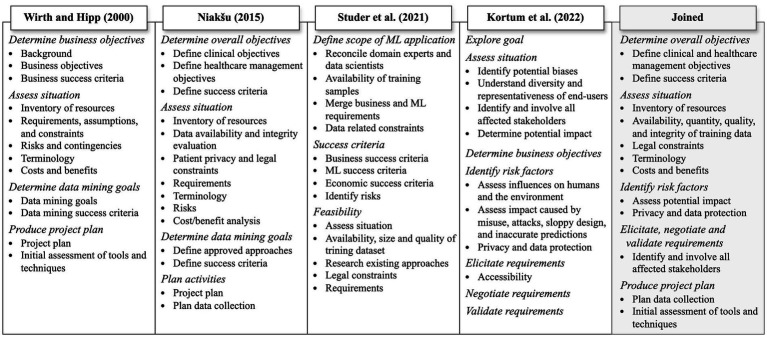
Comparison and combination of steps within the *Business Understanding* phase across different CRISP-DM variants.

In KardioInterakt, the *Business Understanding* phase was initiated during the early stages of project planning to translate the clinically motivated objective of improving chronic HF care into a technically, legally, and ethically viable AI-based telemonitoring system. This entailed defining objectives, assessing the situation, identifying risks, eliciting requirements, and producing an initial project plan (see Figure 3).

The overarching objectives were established at both the clinical and healthcare management levels, with the primary aim of enabling continuous monitoring for the early detection of deterioration. However, inadequate scoping during the preliminary planning stage led to challenges in establishing priorities for subsequent steps (S1: *Unclear Prioritization*). While maintaining flexibility allowed for broad exploration, it also resulted in recurring deliberations regarding system design choices, including sensor technologies (e.g., smartwatches, smart scales), monitored parameters (e.g., NT-proBNP, medication), prediction targets (e.g., mortality, hospitalization), end users (e.g., patients, caregivers), and interaction modalities (e.g., video calls with the attending physician). Success criteria were derived from clinical relevance and technical feasibility, with cardiac decompensation defined as the primary prediction target. Yet, during operationalization, a clinical-technical discrepancy was identified (S2: *Clinical-Technical Misalignment*), as the defined prediction target was not translated into a temporally precise label suitable for modeling, reflecting insufficient alignment between clinical and technical perspectives during problem formulation. This is consistent with prior findings that clinically relevant endpoints are often missing or inadequately documented in healthcare databases (Zemplényi et al., 2023). The situation assessment entailed an evaluation of resources, with a particular focus on time constraints and the availability and quality of data. Initially, project timelines did not adequately account for the lengthy development cycles of medical-grade hardware (S3: *Timeline Underestimation*). Delays in the development of the smart patch constrained the collection of telemonitoring data, necessitating a transition toward routine clinical data and a substantial revision of the project plan. In addition, regulatory constraints (e.g., GDPR compliance and medical device classification) and cost–benefit considerations (e.g., sensor costs and workflow integration) further shaped the system design. A comprehensive risk analysis revealed challenges related to usability, adoption, and safety, including the limited time available to physicians and the reduced technical proficiency among elderly patients. Consequently, the system design was required to minimize user burden and ensure intuitive application, adhering to existing design recommendations (Kortum et al., 2022; Bertl et al., 2023). Additional risks are associated with erroneous predictions, data protection issues, and misuse. Therefore, the system was implemented as a decision-support tool, with strict access and data transmission protocols, and no autonomy in clinical actions. Requirements were elicited by an interdisciplinary team of clinicians and developers. Nevertheless, limited stakeholder diversity was found to be a shortcoming (S4: *Limited Stakeholders*). In accordance with Pinsky et al. (2024) and El Arab et al. (2025), subsequent evaluations highlighted the importance of incorporating additional perspectives, particularly from patients, medical informaticians, ethicists, and legal experts. The lack of legal expertise became especially critical following the introduction of the AI Act, as regulatory and liability concerns could not be adequately addressed within the project team. Thus, an initial project plan was developed but required iterative refinement as new constraints emerged.

### 4.2 Sensor development

**Figure 4:**
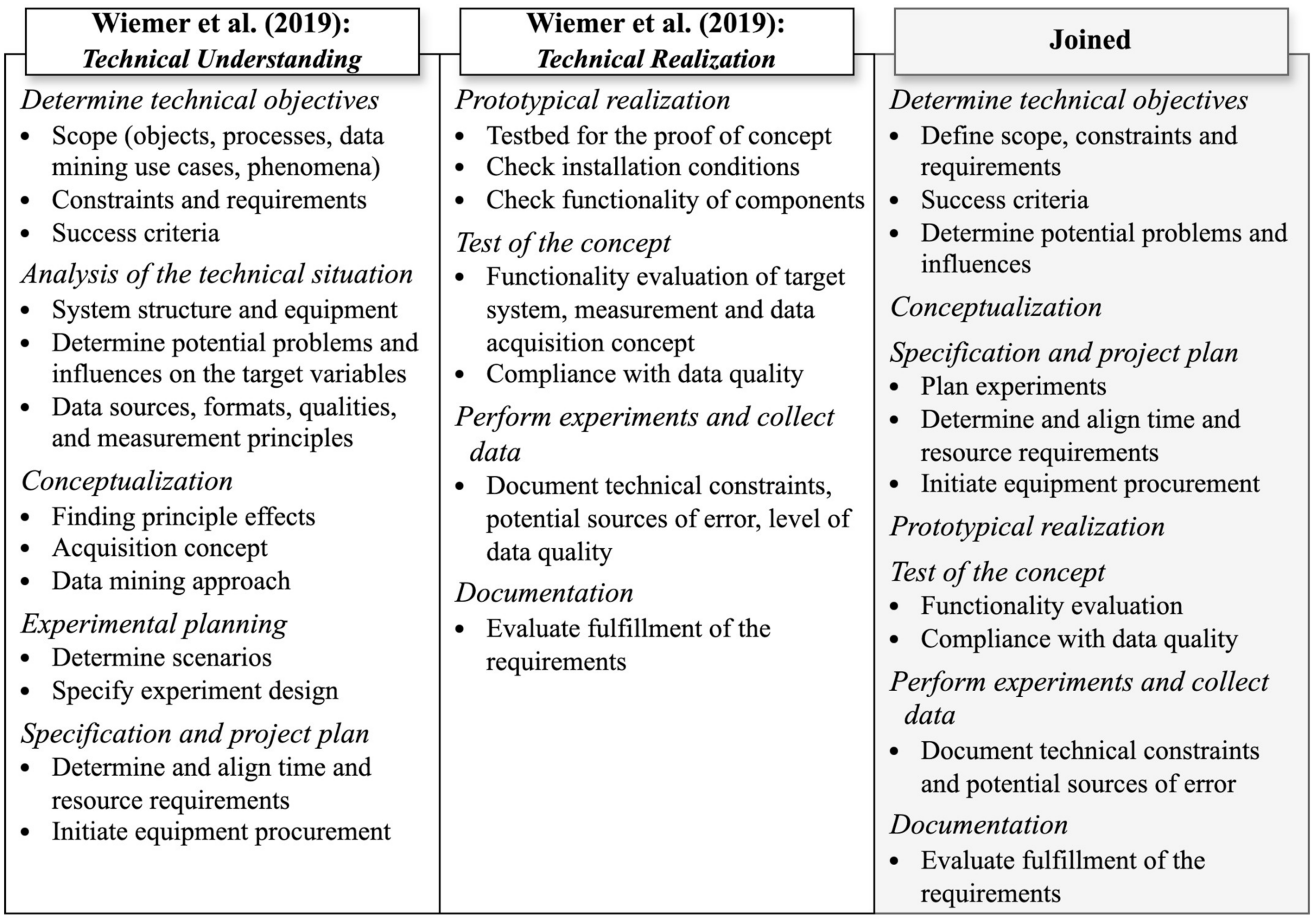
Comparison and combination of steps within the *Sensor Development* phase across different CRISP-DM variants.

The *Sensor Development* phase was dedicated to establishing requirements and success criteria, conceptualizing and prototyping the smart patch, and evaluating its functionality and feasibility within the telemonitoring system. This included specifying technical objectives, conceptualization, development planning, prototypical realization, testing, collecting data, and documentation (see Figure 4).

Technical objectives were defined with respect to usability, measurement capability, and system integration. The smart patch was designed as a lightweight, flexible, and wireless device enabling multiparametric measurements, with data transmission via Bluetooth. To promote sustainability, a two-part architecture was developed, consisting of a disposable patch attached to the skin (containing the foil sensor) and a reusable electronic unit above (encompassing the measuring and transmission unit). Additional design considerations included patient self-application, energy efficiency, and a defined replacement cycle for the disposable component. The development followed a staged approach, commencing with a selection of parameters and point-of-care testing to calibrate sensor signals against laboratory measurements. Conceptualization and prototypical realization revealed the high complexity of developing medical-grade sensor systems. Addressing multiple design dimensions concurrently (e.g., multiparametric measurement, sample volumes, minimal invasiveness, miniaturization, and reusability), led to complex interdependencies and significant development effort (S5: *Design Complexity*), reflecting the inherent complexity of novel sensor development and importance of careful scoping and prioritization (Bandodkar et al., 2016; Heikenfeld et al., 2018). In particular, the measurement intricacy and sensitivity requirements of different biomarkers varied significantly, necessitating parameter-specific design and calibration strategies (Heikenfeld et al., 2018). Consequently, close coordination with clinical laboratories was necessary to ensure the alignment of measurement ranges, reference standards, and sample requirements. The testing of the concept and prototype was constrained by regulatory requirements regarding ethical approvals and medical device certification (S6: *Regulatory Testing Limits*). As patient contact had not been included in the ethics application, end-user evaluations, for instance to ascertain skin type and requirements among elderly patients, could not be conducted. This restricted preliminary assessment of usability aspects under real-world conditions, which is a critical prerequisite for successful integration of the intended system (Bertl et al., 2023; Pinsky et al., 2024). Additional challenges emerged from external dependencies and resource constraints. Supply chain dependencies and procurement timelines required careful planning and buffering (S7: *Supply Chain Dependencies*). In addition, novel sensor technologies can be associated with higher operational costs in the early stages of development, prior to optimization and scaling (Shi et al., 2025), which may limit economic viability and complicate reimbursement (S8: *Cost Burden*). Finally, regulatory requirements, including patenting, validation, and certification as a medical device, yielded complex, time-consuming, and resource-intensive processes (S9: *Regulatory Burden*), despite their integral role in adoption (Bertl et al., 2023).

### 4.3 Data understanding

**Figure 5:**
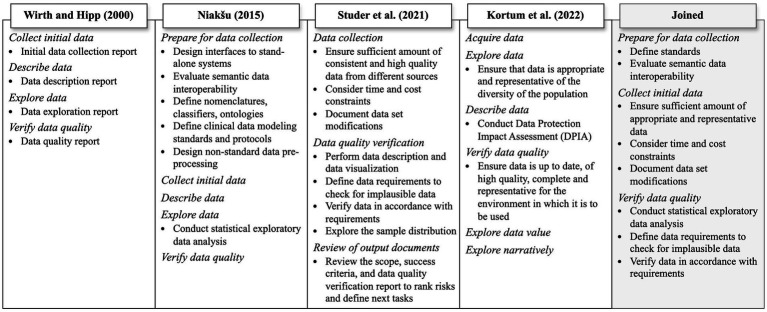
Comparison and combination of steps within the *Data Understanding* phase across different CRISP-DM variants.

The *Data Understanding* phase focused on acquiring, exploring, and assessing data with respect to relevance, quality, and suitability for the intended use case. This encompassed preparation, data collection, exploratory analysis, and quality verification (see Figure 5).

Interdisciplinary collaboration between clinicians and data scientists was essential for establishing a shared understanding of variables and ensuring that data quality met the defined standards in terms of relevance, representativeness, correctness, and completeness, as required for high-risk AI systems under the EU AI Act (European Union, 2024). Due to delays in sensor-based data acquisition, alternative data sources were evaluated. Publicly available datasets for HF telemonitoring were found to be insufficiently aligned with the intended application, either due to missing modalities, incompatible structures or data protection restrictions, which is a prevalent issue in healthcare (Bertl et al., 2023; Zemplényi et al., 2023; Pinsky et al., 2024). Consequently, a dataset of routine checkups comprising 118 patients from a German university hospital was acquired under ethical approval. The dataset comprised laboratory values, diagnostic findings, clinical events, and patient characteristics. A significant challenge emerged during the integration of heterogeneous data sources and the alignment of temporal information (S10: *Temporal Misalignment*), reflecting well-documented interoperability issues in healthcare data environments (Kelly et al., 2019). Due to the unanticipated modification of the project plan, the data collection process proved to be more resource-intensive than anticipated, requiring coordination across multiple stakeholders, including clinicians, medical informaticians, sensor manufacturers, and AI developers (S11: *Coordination Burden*). The exploratory analysis revealed several shortcomings regarding the reliability and validity of the data, a common challenge in healthcare (Zemplényi et al., 2023; Nair et al., 2024). Firstly, the dataset exhibited substantial heterogeneity across patient subgroups (e.g., patients with left ventricular assist devices), which required differentiation to avoid skewed model interpretations (S12: *Data Heterogeneity*). Secondly, a “contextual trap” was identified, wherein technical representations (i.e., current inpatient status) did not align with the clinical prediction objective (i.e., previous hospitalization for decompensation), resulting in spurious correlations (S13: *Contextual Mismatch*). Thirdly, substantial missingness and low measurement frequency impaired the suitability for time-series modeling (S14: *Sparse Data*), consistent with prior findings on typical clinical data limitations (Lin and Haug, 2006; Zemplényi et al., 2023). Furthermore, the target variable (i.e., decompensation status) was only available at the case level without precise temporal annotation (S15: *Temporal Ambiguity*), thereby confirming the previously identified mismatch between the clinical objective and its representation in the data (see S2). An additional challenge was posed by the pronounced class imbalance (S16: *Class Imbalance*), as the number of patients without significant deterioration substantially exceeded those with decompensation events. This is indicative of a prevalent characteristic of clinical datasets, wherein adverse events are comparatively rare and can result in skewed model predictions if not addressed appropriately (Theodorou et al., 2025). Data plausibility issues (e.g., implausible oxygen saturation values) further raised concerns regarding data reliability (S17: *Plausibility Issues*). Finally, certain variables (e.g., spiroergometry results) were not transferable to a telemonitoring context (S18: *Nontransferable* Var*iables*), thereby rendering the information unsuitable for the defined objective (Pinsky et al., 2024). Given these limitations, the routine checkup dataset was deemed inadequate as a basis for model training and validation. Instead, a representative synthetic telemonitoring dataset was constructed based on the routine checkup dataset, clinical guidelines (e.g., German Medical Association et al., 2023) and domain knowledge. A more detailed description of the synthetic data generation process is available in Section 1 of the Supplementary material. Consequently, the subsequent modeling and evaluation efforts merely served as a proof of concept to demonstrate technical feasibility, rather than to establish clinical validity or real-world performance.

### 4.4 Data preparation

**Figure 6:**
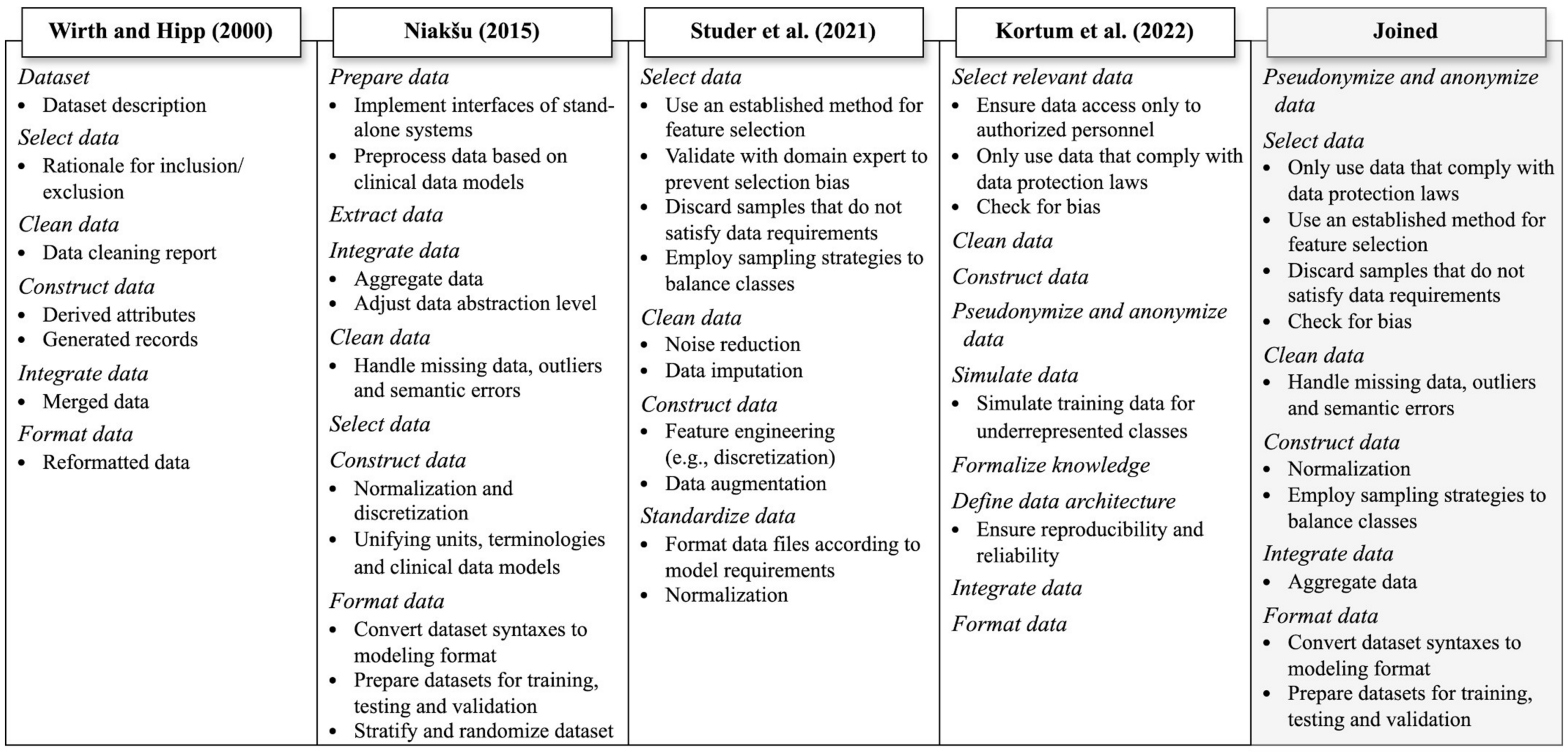
Comparison and combination of steps within the *Data Preparation* phase across different CRISP-DM variants.

The *Data Preparation* phase entailed the construction of a modeling-ready dataset through pseudonymization, selection, cleaning, construction, integration, and formatting of the data (see Figure 6). In the healthcare domain, this phase is particularly intricate due to regulatory, clinical, and technical constraints.

A central consideration was data protection. The collected data were pseudonymized, as telemonitoring requires longitudinal linkage across time and sources. However, the decision between anonymization and pseudonymization proved non-trivial, due to conflicting analytical utility and regulatory compliance (S19: *Privacy Tradeoff*), particularly under varying regional regulations (Pinsky et al., 2024). Pseudonymized data remain subject to stricter regulatory requirements, particularly regarding consent, data sharing, and cross-institutional use. A modular preprocessing pipeline was developed to systematically evaluate different strategies for detecting outliers, handling missing data, feature selection, scaling, and class imbalance (see Section 2 of the Supplementary material). This iterative approach aligns with established best practices in healthcare data preprocessing (Idri et al., 2018; Benhar et al., 2020). However, this process revealed that data preparation is not purely technical but rather a continuous negotiation between regulatory compliance, clinical plausibility, and modeling requirements (S20: *Preparation Tradeoffs*). Handling missing data posed a particular challenge due to significant temporal gaps, for which standard imputation techniques proved to be ineffective. Additionally, clinical stakeholders voiced reservations regarding specific preprocessing techniques, such as imputation and feature selection (S21: *Preprocessing Reservations*), particularly due to concerns about data integrity, underscoring the necessity for interdisciplinary alignment and transparency. Furthermore, our findings suggest that in instances where manual labeling is required, the associated effort must be explicitly considered in resource planning (S22: *Labeling Effort*). Another notable challenge throughout the *Data Preparation* pertains to the limited methodological transparency in existing literature concerning preprocessing pipelines (S23: *Superficial Reporting*). Decisions such as whether to perform imputation and scaling at the patient level or across the entire dataset, whether class balancing should be restricted to the training data, and how to avoid distributing data from the same patient across splits are often insufficiently reported, despite their critical impact on data leakage and model validity (Studer et al., 2021).

### 4.5 Modeling

**Figure 7:**
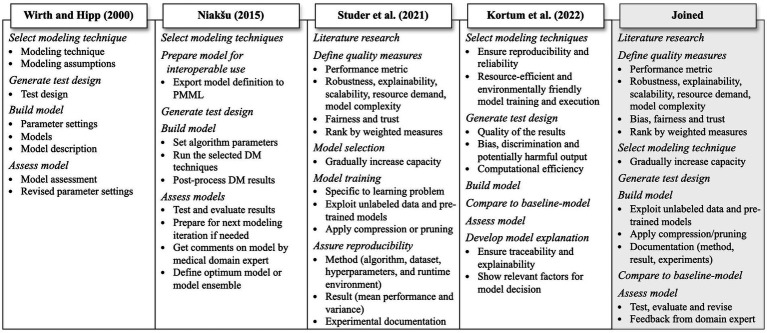
Comparison and combination of steps within the *Modeling* phase across different CRISP-DM variants.

The *Modeling* phase concentrated on the selection, training, and comparison of machine learning models for predicting the risk of cardiac decompensation (see Figure 7). Multiple algorithms were assessed, including tree-based methods and neural networks for sequential data, which are representative of prevalent approaches in HF prediction (e.g., Eid et al., 2021; Yu et al., 2022; Kerexeta et al., 2023).

A set of complementary performance metrics (e.g., accuracy, F1-score, AUC) was selected to enable a more comprehensive assessment. Conversely, existing literature often prioritizes technical performance metrics, while aspects of fairness are scarcely documented (S24: *Fairness Neglect*), as criticized by Kelly et al. (2019). Even when fairness metrics are considered, they may not adequately capture the complexity of real-world diversity and systemic biases inherent in healthcare data (El Arab et al., 2025). The model development followed an iterative approach, gradually increasing model complexity and comparing alternatives (see Section 3 of the Supplementary materials). Nevertheless, methodological guidance from prior work proved limited, particularly regarding hyperparameter tuning and experimental design (S25: *Methodological Uncertainty*), reflecting a broader issue of insufficient reporting transparency in AI research. Another challenge pertains to the definition of clinically meaningful output formats and alert thresholds (S26: *Output Ambiguity*), which are contingent on user preferences and workflow integration (Bertl et al., 2023; Pinsky et al., 2024). Some physicians preferred risk predictions expressed as percentages, while others favored categories. This emphasizes the intricate relationship between modeling decisions and their subsequent application in clinical practice.

### 4.6 Evaluation

**Figure 8:**
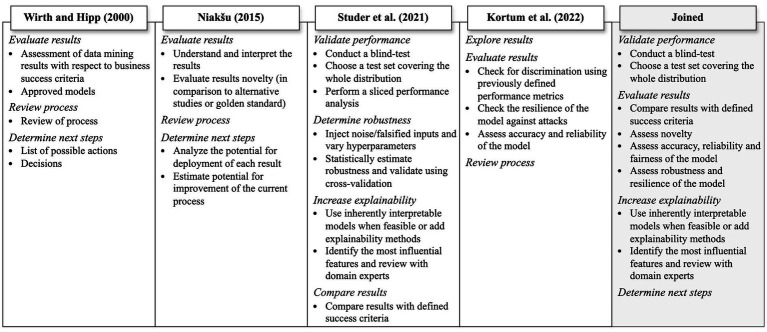
Comparison and combination of steps within the *Evaluation* phase across different CRISP-DM variants.

The *Evaluation* phase assessed whether the developed models met the defined objectives and were suitable for clinical application. This included performance validation, evaluation of results, ensuring explainability, and determining the subsequent steps (see Figure 8).

The performance validation was conducted using a patient-level data split to prevent information leakage. In the proof-of-concept setting, recurrent neural networks demonstrated high predictive performance on simulated telemonitoring data, thereby substantiating their technical feasibility. However, the employment of synthetic data likely imposed limitations on the generalizability of the results and induced overfitting. External validation using real-world telemonitoring data or benchmark datasets was not possible within the scope of the project, as suitable datasets matching the intended use case modalities and temporal structure were not available. In terms of innovation, the project contributes a novel integration of emerging sensor technologies, such as smart patch-based monitoring, into AI-supported telemonitoring pipelines, thereby extending existing approaches that predominantly rely on conventional vital sign monitoring (Kerexeta et al., 2023). Components of the telemonitoring system, including the user interface and AI explanation formats, were evaluated with end users. However, given the proof-of-concept nature of the system, the predictive performance of the AI model and the system as a whole were not evaluated in routine clinical use. A significant constraint in prevailing evaluation practices pertains to the lack of real-world validation involving end users, despite its recognized importance for successful implementation and for assessing potential long-term effects, such as overreliance on AI systems (Bertl et al., 2023; Pinsky et al., 2024). In practice, however, access to relevant stakeholders is often limited, due to the time constraints experienced by clinicians and the difficulty in recruiting and retaining patients for study participation, which may also require explicit inclusion of patient contact in ethics applications (S27: *Stakeholder Access*). Moreover, we found that evaluation in real-life scenarios may not be feasible due to regulatory constraints, which hinder longitudinal testing of the system in routine use (S28: *Regulatory Evaluation Limits*). Another barrier to clinical acceptance appeared to be the limited transparency of complex models (S29: *Limited Transparency*), consistent with prior findings (Petch et al., 2022). Therefore, the explainability of the model was addressed using established XAI methods, including SHapley Additive exPlanations, Counterfactual Explanations, and Anchors (Lundberg and Lee, 2017; Wachter et al., 2017; Ribeiro et al., 2018). Ultimately, the evaluation cycles in healthcare AI are inherently lengthy due to the need for clinical validation and regulatory approval (S30: *Lengthy Validation Cycles*), as model predictions must be medically validated and confirmed through dedicated studies (Pinsky et al., 2024). Consequently, the KardioInterakt project remains at the proof-of-concept stage, with additional validation planned upon the availability of real-world data.

### 4.7 Deployment

**Figure 9:**
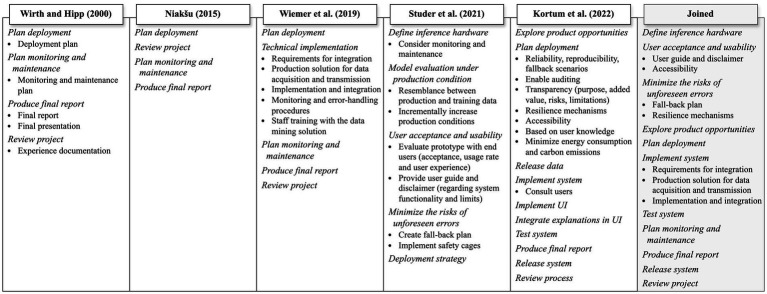
Comparison and combination of steps within the *Deployment* phase across different CRISP-DM variants.

The *Deployment* phase addressed the integration of the system into clinical workflows, usability, minimization of risks, exploring product opportunities, and project review (see Figure 9). As KardioInterakt was developed and evaluated using synthetic data, deployment in a real-world clinical environment was not pursued. Nevertheless, the aspects of the phase were conceptualized and incorporated into the system design.

A central focus was on the processes of onboarding users and fostering their acceptance. To ensure safe and effective use, structured onboarding materials were developed, including an in-app tutorial, detailed background information, and self-assessment. Concurrently, a lack of established standards for onboarding materials regarding AI systems in healthcare was identified (S31: *Onboarding Gap*), despite its importance for successful integration of AI (Cai et al., 2019; Lambert et al., 2023). Ethical, legal, and social implications (ELSI) emerged as critical yet frequently under-addressed aspects of high-risk system deployment (S32: *ELSI Underaddressed*), particularly in the context of AI-supported patient care, where issues such as privacy, safety, trust, and responsible clinical decision-making are directly affected (Gerke et al., 2020; Reddy and Shaikh, 2025). In particular, regulatory requirements such as the EU AI Act and medical device regulations introduce significant complexity. In accordance with Nair et al. (2024), we found that inadequate legal expertise impedes decision-making processes concerning liability and system classification (S33: *Legal Expertise Gaps*), while prolonged approval processes and the necessity of substantial initial investments further compound the challenges (S34: *Approval Delays*). Our observations indicate that technical deployment is further complicated by the fragmented healthcare IT landscape, which limits interoperability with existing systems (S35: *Interoperability Barriers*), as also noted in prior work (Bertl et al., 2023). Hence, the KardioInterakt system was conceived as a standalone app- and web-based solution with import and export functionalities to facilitate integration with existing clinical systems. Finally, the transferability of solutions to other use cases is often insufficiently considered (S36: *Limited Transferability*), despite its importance for scalable and sustainable healthcare AI systems (Pinsky et al., 2024; El Arab et al., 2025). Therefore, we explicitly assessed transferability, concluding that the developed system may prospectively be applicable to other chronic diseases requiring continuous monitoring, such as diabetes or liver insufficiency.

### 4.8 Monitoring and maintenance

**Figure 10:**
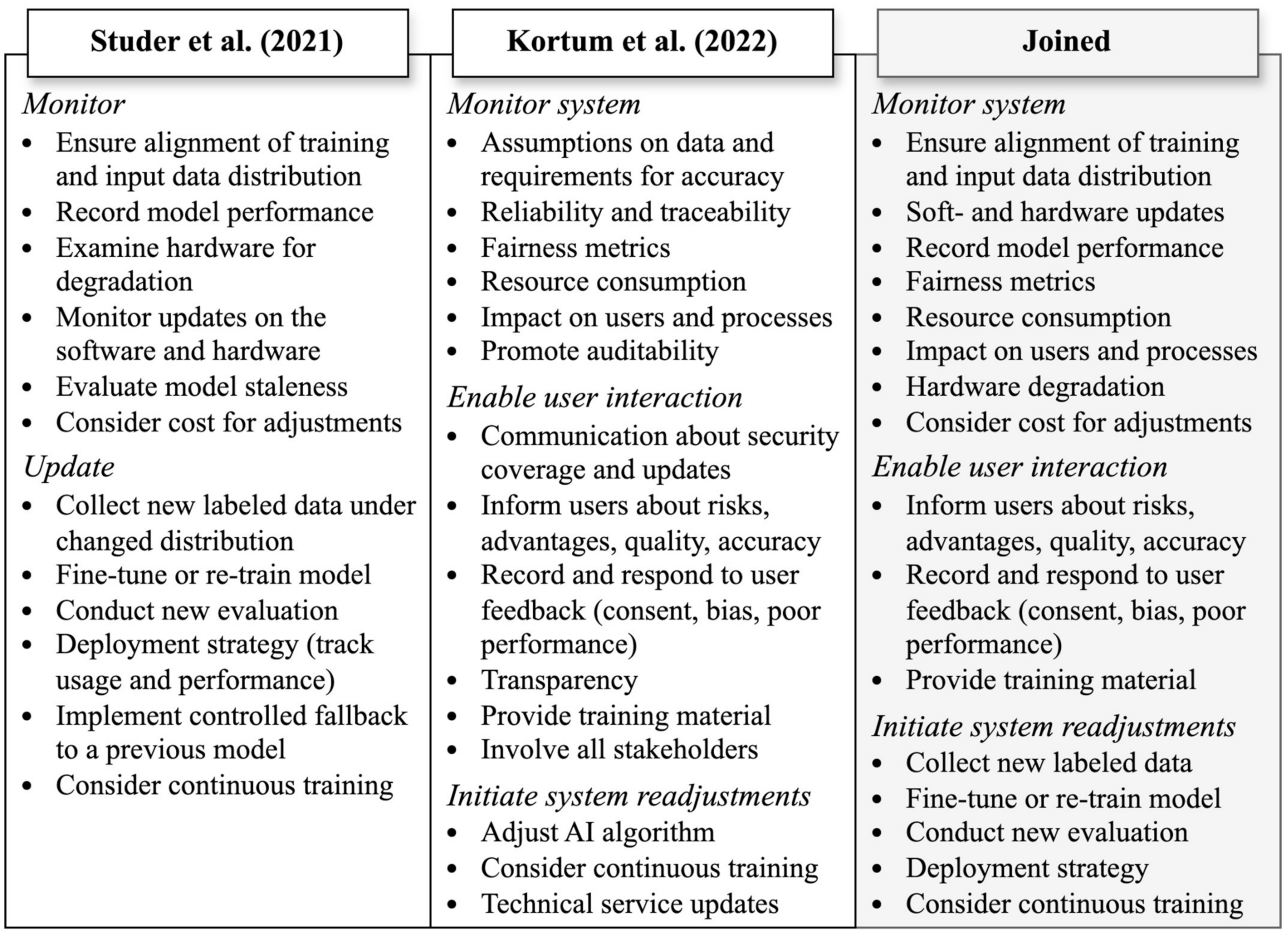
Comparison and combination of steps within the *Monitoring and Maintenance* phase across different CRISP-DM variants.

The *Monitoring and Maintenance* phase focused on ensuring sustained model performance, safety, and alignment with clinical practice over time (see Figure 10). In alignment with the proof-of-concept setting, requirements and design considerations were addressed at a conceptual level to enable prospective real-world operation.

A key prerequisite is the alignment between training data and real-world input data, which will be achieved through the acquisition of telemonitoring data from the novel smart patch technology. To ensure sustained model performance, mechanisms for collecting and responding to user feedback were implemented and emphasized during onboarding. These mechanisms enabled users to report both usability issues and discrepancies between their own medical assessment and model predictions. Moreover, users were made aware of system functionality, limitations, and consequences of use in addition to being provided with further training materials, as recommended in the literature on onboarding and user training for AI systems in healthcare (Cai et al., 2019; Garvey et al., 2022). In this context, user interaction constitutes a core component of this phase. Systems must be adapted to the needs of different user groups (S37: *User Adaptation Needs*), such as elderly patients with limited technical literacy and time-constrained clinicians who are averse to disruptive alerts (Kortum et al., 2022; Bertl et al., 2023). Another major challenge pertains to the resources necessary for system readjustments through continuous training (S38: *Retraining Resources*). This is particularly pronounced in scenarios where true labels for new data are not automatically available and must be manually added, as approaches such as active learning require dedicated infrastructure and verification effort by experts. Beyond model updates, system maintenance in telemonitoring contexts also includes ensuring reliable operation of medical devices, regular software updates, and monitoring for hardware degradation (Studer et al., 2021; Kortum et al., 2022). We found that this requires long-term planning of time, cost, and personnel (S39: *Long-Term Planning*), which is often underestimated in AI projects. The rapid evolution of AI methods and wearable technologies necessitates continuous adaptation, yet responsibilities for maintaining and updating such systems are not always clearly delineated, particularly following the transition from research to operational settings.

### 4.9 Cross-phase synthesis of symptoms, obstacles, causes, and mitigation strategies

The preceding phase-based analysis identified recurring symptoms, consolidated obstacles, and underlying causes, across the development lifecycle. Table 2 synthesizes these relationships across phases and complements them with derived mitigation strategies. The symptom identifiers in brackets following the proposed project solutions indicate how these mitigation strategies map to the respective challenges. While the causes, obstacles, and symptoms reflect the empirical case analysis, the mitigation strategies represent an interpretive synthesis informed by the project experience and, where applicable, related literature.

**Table 2:** Overview of causes, obstacles, symptoms, and mitigation strategies within the development process for AI-based telemonitoring systems.

ID	Symptoms	Obstacles	Causes	Project solution	Aspirational solution
1	*Unclear Prioritization* (S1), *Timeline Underestimation* (S3), *Design Complexity* (S5), *Supply Chain Dependencies* (S7), *Labeling Effort* (S22), *Lengthy Validation Cycles* (S30), *Retraining Resources* (S38), *Long-Term Planning* (S39)	Scope creep, ambitious timeline and interdependencies	Limited prior expertise and funding	Narrow the project to one high-value use case (S1, S3, S5); include time, budget, compute, and data buffers (S3, S7, S22, S30, S38); decide early whether core components should be built internally or procured externally (S3, S5), anticipate long-term operational requirements early (S39)	Longer funding periods and centers of excellence to build and reuse AI infrastructure and expertise across projects
2	*Limited Stakeholders* (S4), *Coordination Burden* (S11), *Stakeholder Access* (S27), *Legal Expertise Gaps* (S33)	Limited availability and involvement of all relevant stakeholders	Underestimated complexity of AI-based telemonitoring system development; inadequate funding	Involve missing stakeholder groups earlier and more systematically (S4, S27, S33); secure input from clinicians, patients, technical experts, and governance actors (S4, S27, S33); define collaboration checkpoints (S11, S27, S33)	Establish formal multi-stakeholder development structures as standard practice, including medical associations and patient representation
3	*Clinical-Technical Misalignment* (S2), *Contextual Mismatch* (S13), *Sparse Data* (S14), *Temporal Ambiguity* (S15), *Nontransferable Variables* (S18)	Data not aligning with the specific requirements of the intended application	Insufficient early data audit and feasibility assessment	Conduct an early feasibility and data-quality audit (S2, S13, S14, S15, S18); derive data requirements directly from the use case (S13, S14, S18); align technical labels, variables, and outcomes with the underlying clinical question (S2, S13, S15); ensure close physician-developer coordination (S2, S13)	Design data collection infrastructures prospectively around clinical AI use cases rather than relying on opportunistic secondary data
4	*Temporal Misalignment* (S10), *Data Heterogeneity* (S12), *Class Imbalance* (S16), *Plausibility Issues* (S17), *Preprocessing Reservations* (S21)	Characteristics of clinical data that impede AI-based analysis and interoperability	Workflow-data misalignment; ambiguous clinical ground truth; inconsistent documentation	Clarify the intended target condition and decision context (S10, S12, S16); jointly inspect data across disciplines to identify documentation gaps and ambiguous ground truth (S10, S12, S17, S21); define interoperability requirements early (S10)	Promote standardized documentation, interoperable data architectures, and clearer clinical labeling conventions across institutions
5	*Regulatory Burden* (S9), *Privacy Tradeoff* (S19), *Preparation Tradeoffs* (S20), *Approval Delays* (S34)	Cumbersome legal classification and clarification of liability issues, lengthy and expensive processes	Complicated, extensive and vague regulatory landscape; sponsoring required; missing operational acceptance criteria	Involve legal and regulatory expertise from the beginning (S9, S19, S20, S34); clarify the legal basis for data acquisition and system classification early (S9, S19, S34); define operational acceptance criteria before development progresses too far (S20, S34)	Develop more specific, actionable regulatory guidance and clearer liability frameworks for AI-based telemonitoring applications
6	*Regulatory Testing Limits* (S6), *Cost Burden* (S8), *Output Ambiguity* (S26), *Regulatory Evaluation Limits* (S28), *Limited Transparency* (S29), *Onboarding Gap* (S31), *Interoperability Barriers* (S35), *User Adaptation Needs* (S37)	Development out of touch with the everyday lives of those affected	Strict regulatory requirements; limited availability of end-users; fragmented IT landscape hinders universal integration	Test the system with real users in realistic care settings (S26, S29, S31, S37); adapt implementation and onboarding to existing routines (S6, S26, S28, S31, S35, S37); use explainability features where they support trust and usability (S26, S29, S37); assess cost, scalability, and reimbursement early (S8)	Create better conditions for routine end-user participation; improve cross-system integration within fragmented healthcare IT environments
7	*Superficial Reporting* (S23), *Fairness Neglect* (S24), *Methodological Uncertainty* (S25), *ELSI Underaddressed* (S32), *Limited Transferability* (S36)	Insufficient publication of unsuccessful efforts and superficial method reporting hinder learning from previous efforts	Positive publication bias; (medical) datasets not publicly available	Report failed experiments, dead ends, and design revisions more explicitly (S23, S25); document methods, preprocessing steps, and modeling choices in enough detail for replication (S23, S25, S36); report not only technical but also fairness, and ELSI aspects (S23, S24, S25, S32); reuse solutions and assess transferability (S23, S25, S36)	Increase acceptance of negative results; adopt reproducibility-oriented publication formats; facilitate accessibility and provision of shareable clinical benchmark datasets

## 5 Discussion

### 5.1 Implications for practice

*Scope conservatively and plan for feasibility early*: Within the *Business Understanding* phase, AI-based telemonitoring projects should define a sharply bounded use case from the outset and avoid pursuing an overly broad solution space in early stages (Lanotte et al., 2023; Li et al., 2023). In regulated healthcare contexts, this is particularly important because vague objectives can amplify downstream complexity in data, infrastructure, validation, workflow integration, and regulatory clarification. Our findings further suggest that planning should rely on conservative estimations of time, effort, and organizational readiness. Projects should account for delays, coordination overhead, limited internal know-how, and the possibility that assumptions about data, hardware, or implementation effort prove too optimistic. This also highlights the need for early anticipation and contingency planning around data availability and technical infrastructure, since fragmented data environments and limitations in digital infrastructure can create major downstream barriers for AI implementation (Nair et al., 2024). In addition, make-or-buy decisions should be addressed early, particularly regarding expected cost and time commitments (Hart et al., 2023). Furthermore, ambitious healthcare AI initiatives appear to benefit from phased, pilot-based development rather than full end-to-end realization in one step, as smaller milestones allow clearer feasibility checks and decision points before proceeding further (Hammer et al., 2025). Finally, our findings suggest that current governance and funding structures may not adequately reflect the high time and cost requirements of healthcare AI development, consistent with similar observations by Davat et al. (2024). Because substantial resources are needed for data preparation, infrastructure, and interdisciplinary coordination, AI-related capacities should not be built for a single project only, but developed, reused, and continuously expanded across projects, for example through shared support structures or centers of excellence that can realize economies of scale (Zemplényi et al., 2023).

*Broaden stakeholder involvement and structure collaboration early:* A key recommendation is to ensure the early and continuous involvement of a broad and relevant set of stakeholders throughout development, since restricted availability and narrow participation can weaken coordination, delay decisions, and reduce alignment between clinical, technical, organizational, and regulatory requirements. This is already relevant in the *Business Understanding* phase, where the involvement and thus funding of the necessary stakeholders should be considered from the outset. Because these systems sit at the intersection of clinical practice, technical development, organizational routines, patient needs, and regulation, recommended stakeholders include an interdisciplinary group of clinicians, AI and IT experts, patients, and regulatory expertise (Mbunge et al., 2021; Alami et al., 2024). However, the recruitment of such experts should be treated as a central project-management task rather than an implicit precondition, since it can become a major limitation when time and resources for participation are scarce (Davat et al., 2024). Beyond mere participation, projects should also establish clear responsibilities and defined collaboration milestones and, where internal capacities are limited, consider external partnerships or sourcing options at an early stage (Hart et al., 2023; Nair et al., 2024).

*Align data strategy early with the intended use case*: Data acquisition and the assessment of requirements of the intended application should be designed from the outset, because insufficiently audited or weakly aligned data can create a mismatch between the clinical objective and the technical solution, thereby undermining feasibility, model validity, and later practical usefulness. A key recommendation is therefore to conduct early and rigorous data audits before substantial development resources are committed (Chomutare et al., 2022). To further reduce risk, projects should audit data early to assess feasibility for the intended use (Zemplényi et al., 2023). Achieving this alignment requires close collaboration between developers and physicians, since only interdisciplinary exchange can ensure that clinically meaningful questions are translated into technically appropriate data structures and model inputs (Polevikov, 2023; Alami et al., 2024).

*Explore clinical data deeply and plan interoperability early*: Another key recommendation for AI-based telemonitoring initiatives is to deeply explore available data early, to ensure clinical utility (Golden et al., 2024) of available data, and estimate the effort of aligning the clinical meaning of variables, documentation practices, and technical representations (Wen et al., 2024). This is particularly important in healthcare, where data are often heterogeneous, differently structured across systems, and only partially suited for direct analytical use (Kelly et al., 2019). In addition, projects should plan explicitly for interoperability and integration rather than treating these issues as downstream technical tasks. Prior work shows that successful implementation depends on interoperability with hospital systems and on the ability to align variables, formats, and terminologies across data sources (Chomutare et al., 2022). Taken together, these findings suggest that challenges related to clinical data characteristics cannot be addressed through technical preprocessing alone, but require early scoping, interdisciplinary interpretation, and proactive planning for integration into fragmented healthcare IT environments.

*Clarify legal requirements early and involve legal expertise:* Legal and regulatory issues should be treated as core development challenges rather than downstream administrative tasks, since unresolved questions around classification, liability, and lawful data use can affect timelines and costs (Reddy and Shaikh, 2025). This is particularly relevant in healthcare AI, where legal classification, approval requirements, data protection obligations often intersect and remain only partially operationalized in practice (Li et al., 2023). Projects should therefore treat early system classification as an explicit development activity, since the applicable regulatory pathway depends on whether the intended AI application falls under medical device and related high-risk regulatory frameworks (Zsidai et al., 2023; Wen et al., 2024). Thus, a key recommendation is to translate broad legal requirements into project-specific and operational acceptance criteria, so that legal and governance requirements are translated into concrete development targets, responsibilities, and approval conditions rather than remaining abstract compliance concerns (Hart et al., 2023; Reddy and Shaikh, 2025). In addition, projects should collaborate closely with legal and data-governance experts to address regulatory concerns and liability (Alami et al., 2024). The same applies to the legal basis for data acquisition and processing, including informed consent, data protection protocols, and the use of safeguards such as anonymization or pseudonymization (Ueda et al., 2024). Consequently, our findings suggest that regulatory compliance in AI-based telemonitoring should not be handled reactively but planned proactively as a core part of system development.

*Evaluate in real-world settings and support adoption through explainability and onboarding*: Development can easily become detached from the clinical and patient reality when regulatory constraints on patient access and fragmented IT environments prevent seamless integration into routine care. To mitigate this risk, systems should be evaluated with real end-users in realistic usage scenarios rather than only under controlled technical conditions. Prior work emphasizes the importance of aligning AI implementation with existing workflows, piloting software against standard practice, and incorporating user feedback before and during rollout (Siira et al., 2024). Such real-world evaluation helps identify usability, workflow, and acceptance problems that may remain invisible in purely technical assessments. In addition, adoption should be supported through context-appropriate explainability and structured onboarding (Petch et al., 2022; Lambert et al., 2023). Explainability needs to be adapted to the audience and use context, since transparency and interpretability are important conditions for trust and effective use in practice (Schouten et al., 2022). At the same time, onboarding should not be treated as an informal afterthought, but as a defined implementation process that includes training, educational materials, and opportunities for hands-on experience and feedback (Schouten et al., 2022). Taken together, these findings suggest that successful AI-based telemonitoring depends not only on technical performance, but also on whether systems are tested, explained, and introduced in ways that fit clinical and organizational environments.

*Report unsuccessful efforts and methods more transparently:* Progress in this field depends not only on successful model development, but also on the field’s ability to learn from unsuccessful or only partially successful efforts. Therefore, a key proposition is to increase the acceptance of so-called negative publications and to report experiments, methodological choices, and development conditions in substantially greater detail (Stanley, 2005). Similarly, prior work suggests that while open-access healthcare datasets can promote transparency, reproducibility, and collaboration, such datasets remain limited and are often restricted to one or a few institutions, underscoring the need for more shareable and diverse clinical benchmark datasets (Alberto et al., 2023). When datasets are not publicly available and publications focus mainly on successful outcomes, learning from prior work becomes difficult, and superficial reporting further limits the transferability of findings to new projects. More detailed reporting is particularly important for understanding why specific approaches did not work, which assumptions proved unrealistic, and under which technical, clinical, or organizational conditions results were obtained. This includes reporting not only final model performance, but also data limitations, unsuccessful experiments, design trade-offs, stakeholder involvement, and implementation constraints. Implicit ethical concerns should likewise be reflected and reported, including less immediate or downstream consequences such as sustainability considerations or the risk of overmedicalizing end-of-life care (Davat et al., 2024). Such transparency may improve traceability, support cumulative learning across projects, and reduce the risk that future teams repeat avoidable mistakes. In this sense, better publication practices are not merely an academic ideal, but a practical requirement for more robust and transferable AI development in healthcare.

### 5.2 Implications for research

*Rebalance methodological attention toward the early phases of AI development:* AI-based telemonitoring should not be framed primarily as an analytical challenge centered on modeling performance. Our findings suggest that the early phases from *Business Understanding* to *Data Understanding* are particularly decisive for project success in regulated healthcare contexts. Many of the obstacles identified in this study emerged before model development in the narrower sense, for example in the definition of the target use case, stakeholder involvement, feasibility assessment, clarification of regulatory requirements, and the alignment between clinical questions and available data. Prior work likewise suggests that difficulties in these early phases can generate downstream problems for implementation, adoption, and scale-up in healthcare AI (Chomutare et al., 2022). This indicates that future research should place stronger conceptual and methodological emphasis on these upstream phases rather than treating them merely as preparatory work for later analytical stages. In this sense, healthcare AI development is not only a data-scientific task, but a socio-technical and regulatory design problem whose success is often determined before the *Modeling* phase begins.

*Stronger integration of implementation science and translational perspectives into AI development:* Our observations further suggest that AI-based telemonitoring should not be conceptualized as a technical development process that is only later translated into practice. We found that many of the most relevant obstacles emerge precisely at the interface between development, implementation, and translation into real-world care, for example in workflow alignment, stakeholder coordination, interoperability, onboarding, and the practical interpretation and use of AI outputs. Prior work has highlighted the need to move beyond a linear view of AI development and to address implementation, workflow fit, local context, and real-world impact as integral parts of system design rather than downstream concerns (Coiera, 2019). This indicates that future research should integrate implementation science and translational perspectives much earlier into AI development research and treat translation not as a downstream step after model completion, but as a condition that shapes system design from the outset. For regulated healthcare AI, this may help generate process models and evaluation approaches that better reflect the realities of adoption, integration, and sustained clinical use.

*Move CRISP-DM research toward modular and domain-sensitive adaptation*: Shimaoka et al. (2024) show that CRISP-DM has been repeatedly adapted across a wide range of contexts through phase additions, phase modifications, integrations with other methods, and the addition of supporting tools and features. This suggests that the enduring value of CRISP-DM lies less in its fixed original formulation than in its adaptability as a reference structure. Our findings support this view but also indicate that healthcare AI telemonitoring requires a particularly demanding combination of extensions, including explicit attention to clinical validity, regulatory requirements, deployment constraints, and post-deployment monitoring. Rather than proposing a completely new process model, our synthesis demonstrates the value of combining CRISP-DM with complementary variants to create a domain-sensitive lifecycle model. Future research may therefore benefit from moving toward more modular CRISP-DM architectures that preserve the core logic of the model while allowing context-specific phases and tasks to be added for settings such as sensor-based telemonitoring, high-risk regulation, and long-term human oversight.

*Extend CRISP-DM research to data-generating and sensor-based AI systems:* Projects in AI-based telemonitoring often rely on data that are not simply available at the outset, but are shaped by upstream decisions on device design, measurement engineering, and infrastructure development (Ates et al., 2022). Our findings suggest that in AI-based telemonitoring, sensor development constitutes a distinct methodological challenge because it directly influences what data can be collected, with what quality, under which practical conditions, and with which implications for later modeling, evaluation, and deployment. This differs from many classical data-mining settings, in which the central task is to analyze and transform a collected dataset rather than to design the sensing and data-generating infrastructure itself (Wirth and Hipp, 2000). Future CRISP-DM research may therefore benefit from addressing more explicitly those contexts in which data generation itself is a core part of the development process. For telemonitoring and related healthcare AI applications, this implies a stronger process-level integration of sensing infrastructure, data acquisition conditions, and operational constraints.

### 5.3 Limitations and future work

Several limitations should be considered when interpreting the findings. First, the study is based on a single qualitative case in a specific German research and regulatory context, which limits the transferability to other healthcare domains, organizational settings, or national environments. At the same time, the case is grounded in a three-year project with continuous exchange among diverse stakeholders, yielding rich empirical insights and a strong basis for evaluating the derived recommendations intended to inform the development of similar AI-based telemonitoring systems. Future research should examine additional cases across different clinical contexts and regulatory settings to assess the generalizability of the identified obstacles and mitigation strategies. Additionally, these process-level findings should be examined further during validation with prospective real-world telemonitoring data. Second, the analysis is partly retrospective and interpretive, meaning that the reconstruction of obstacles, causes, and mitigation strategies may be influenced by hindsight and by the perspectives available within the project. This could be complemented by prospective or longitudinal study designs. Third, the proposed framework was not externally validated, thus while it is grounded in the present case, its applicability to other settings should be further examined through application in additional projects. Similarly, the derived mitigation strategies were not systematically validated across multiple cases and should therefore be understood as practice-informed and analytically grounded recommendations rather than universally established best practices. Their robustness could be further assessed through comparative studies, controlled evaluations, or implementation in additional projects. Furthermore, while prior literature was used to contextualize the findings, the paper does not provide a systematic or exhaustive review of all possible development barriers and responses. The study thus contributes process-level insight and transferable orientation, but not definitive or universally generalizable guidance. Comprehensive reviews or meta-analyses could consolidate and extend the evidence base. Finally, the selection of CRISP-DM variants was not based on a systematic review, and the proposed synthesis was not formally evaluated, which may limit completeness and representativeness. Future work could extend this approach through systematic identification and comparative evaluation of suitable process models.

## 6 Conclusion

This paper advances the discussion on AI-based telemonitoring by shifting attention from isolated technical performance to the full development lifecycle. By synthesizing CRISP-DM variants, it identifies the process phases applicable to AI-based telemonitoring and extends them into an eight-phase structure that includes *Sensor Development* and *Monitoring and Maintenance*. Through its application to a real-world case, the study reveals which methodological, regulatory, and practical obstacles and underlying causes arise across this lifecycle. Building on this analysis, actionable mitigation strategies are derived that translate these insights into development guidance. The key innovation lies not only in identifying individual barriers, but in linking symptoms, obstacles, causes, and mitigation strategies across phases within a structured process lens. For researchers, developers, and healthcare organizations, this provides a practical framework with potential transferability for anticipating challenges and developing more robust, trustworthy, and implementation-ready telemonitoring systems.

## Data Availability

The original contributions presented in the study are included in the article/Supplementary material, further inquiries can be directed to the corresponding author.
