# Supplementary material for: Symptoms and causes of obstacles in AI-based telemonitoring: strategies for system development through a CRISP-DM lens
Source: Front Artif Intell. 2026 Jun 26;9:1850272. doi: 10.3389/frai.2026.1850272 (PMC13350275; doi:10.3389/frai.2026.1850272)
Supplement: Supplementary file 1 [file Data_Sheet_1.PDF]

Supplementary Material

1 Synthetic Data Generation

Because the available routine checkup dataset was inadequate for model training and validation, a representative synthetic telemonitoring dataset was generated for proof-of-concept AI development. The synthetic dataset was informed by relevant information from clinical checkup data, medical guidelines, and literature-based insights, but does not claim clinical validity.

The generation process allowed experimental adjustment of the measurement frequency per day, the total observation period, and the number of simulated patients. Measurements were modeled within clinically plausible value ranges. To approximate realistic disease dynamics, baseline states were modeled and followed by controlled improvements or deteriorations, including vital-sign measurements with time-of-day-dependent fluctuations. The dataset also differentiated clinically relevant groups, including decompensation status, sex, and NYHA classification, and included special cases such as fatalities. Subsequently, the synthetic dataset was reviewed by medical experts from the consortium for plausibility and internal consistency.

2 Data Preparation

A modular preprocessing pipeline was developed to evaluate alternative data preparation strategies for the synthetic dataset while ensuring scalability for future real-world data. As the synthetic dataset was generated with complete records, plausible value ranges, predefined features, and balanced scenario representation, the final pipeline did not require outlier handling, missing-value imputation, feature selection, or class balancing. Feature scaling was fitted on the training data and applied downstream to the validation and test set to prevent information leakage between training, validation, and test data. For the same reason, the data was partitioned according to a strict patient-level split. The experimental options and the final choices for the proof-of-concept configuration are detailed in Supplementary Table 1.

Supplementary Table 1. Preprocessing experiments and final configuration.

Component	Experimental Options	Final Configuration
Data split	60/20/20, 70/15/15, 80/10/10 training/validation/test split	70% training, 15% validation, and 15% test data
Outlier detection	None, z-score, interquartile range, local outlier factor, isolation forest	None
Missing value imputation	None, deletion, forward fill, backward fill, mean, most frequent, interpolation, k-nearest neighbors, multivariate imputation by chained equations, expectation maximization, linear support vector regression	None
Feature selection	None, chi-square, ANOVA F-value, mutual information, random forest, linear support vector machine, linear regression coefficients, ReliefF, correlation-based selection	None

Feature scaling	None, min-max scaling, z-score normalization, robust scaling	Min-max
Class balancing	None, random over-sampling, synthetic minority over-sampling technique	None
Sliding windows	Input window: 7, 14, 21 days<br>Forecast horizon: 7, 14, 21 days<br>Window stride: 1	14-day input window and 14-day forecast horizon with stride 1

3 Modeling

Several machine learning models were implemented and compared for predicting decompensation risk, as can be seen in Supplementary Table 2. The recurrent neural network configuration achieved the best proof-of-concept performance, with 0.2 loss, 94% accuracy, 90% precision, 97% recall, 93% F1-score, and 98% AUROC. However, given the reliance on synthetic data, concerns regarding overfitting and limited generalizability are plausible. In the future, clinical validation based on real-world telemonitoring data will be necessary.

Supplementary Table 2. Modeling experiments and final configuration.

Component	Experimental Options	Final Configuration
Dataset	Size: 100, 300, 500 patients<br>Observation period: 30, 60, 90 days<br>Measurement frequency: 1, 3, 5 observations per day	300 patients over 30 days with a single observation per day
PyTorch Model	Random forest, XGBoost, vanilla recurrent neural network, gated recurrent unit, long short-term memory	Vanilla recurrent neural network
Hidden size	8, 16, 32	16
Layers	1, 2, 3	2
Nonlinearity	Tanh, ReLU	Tanh
Dropout	0, 0.1, 0.2	0.1
Bidirectional	True, false	False
Epochs	75 with early stopping	45
Batch size	16, 32, 64	32
Optimizer	Adam, AdamW, stochastic gradient descent	AdamW with 0.0001 learning rate and 0.01 weight decay
Regularization	Gradient clipping, early stopping patience	1.0 gradient clipping norm and 2 early stopping patience
Loss function	Binary cross-entropy with logits	Binary cross-entropy with logits and 1.0 positive-class weight
